# Effect of dietary nonphytate phosphorus on laying performance and small intestinal epithelial phosphate transporter expression in Dwarf pink-shell laying hens

**DOI:** 10.1186/2049-1891-4-34

**Published:** 2013-09-12

**Authors:** Wei Nie, Ying Yang, Jianmin Yuan, Zhong Wang, Yuming Guo

**Affiliations:** 1 State Key Laboratory of Animal Nutrition, College of Animal Science and Technology, China Agricultural University, Beijing 100193, China

**Keywords:** Calbindin, Laying hens, Nonphytate phosphorus, Sodium phosphate co-transporter, Vitamin D receptor

## Abstract

This study examined the effects of various levels of dietary nonphytate phosphorus on laying performance and the expression patterns of phosphorus metabolism related genes in Dwarf pink-shell laying hens. A total of 405 28-week-old Dwarf pink-shell laying hens were fed the same corn-soybean basal meals but containing 0.20%, 0.25%, 0.30%, 0.35% or 0.40% nonphytate phosphorus. The results showed that feed intake, egg production, and average egg weights were quadratically correlated with dietary nonphytate phosphorus content (*P* < 0.05), and the highest egg production, feed intake and average egg weights were achieved when dietary nonphytate phosphorus was at 0.3% (*P* < 0.05). mRNA expression of intestinal sodium phosphorus co-transporter linearly decreased when dietary nonphytate phosphorus increased. mRNA and protein expression of intestinal calbindin and vitamin D receptor correlated quadratically with dietary nonphytate phosphorus, and the highest expression was found when dietary available phosphorus was at 0.25% to 0.3%. In conclusion, the ideal phosphorus requirement for Dwarf pink-shell layer hens is estimated to be 0.3% in a corn-soybean diet. With this level of phosphorus supplementation, calbindin and vitamin D receptor reached their highest expression.

## Introduction

Phosphorus is an essential nutrient for animals. Phosphorus plays numerous roles in nutrient metabolism and is also an essential component of genetic material, membrane phospholipids and bone. There is increasing concern over excess phosphorus pollution in the environment from poultry production. To reduce excess phosphorus without compromising poultry production, an accurate evaluation of the amount of phosphorus required for laying hens needs to be conducted. Levels of available dietary phosphorus may adversely affect laying hens’ production and eggshell quality [1,2]. Dwarf pink-shell laying hens are a layer strain bred by China Agricultural University that have a sex-linked dwarf gene. A normal adult laying hen of this strain weighs about 1,600 g. Laying hens with the dwarf gene are 10 cm shorter than laying hens without the gene [3]. Because of their smaller size, the average daily feed intake during peak egg production is about 85–95 g/bird, 20–25% less than the parent strain, but egg production is increased by 10–15% compared with their counterpart laying hens [3]. It is very likely that the dwarf hen has different phosphorus requirements. So far, little work has been done to assess the phosphorus requirements of Dwarf pink-shell laying hens.

Many factors can regulate phosphorus absorption in the small intestine. The phosphorus transporter, sodium phosphorus IIb (NaP-IIb), is reported to be positively regulated by vitamin D and vitamin D receptor. It seems that NaP-IIb expression or activity could be enhanced through the vitamin D endocrine system [4]. Dietary calcium also has a large impact on phosphorus absorption in the small intestine of chickens [5]. In Ca^2+^-transporting epithelia, calbindin-D28k could act as a Ca^2+^ buffer [6]. It has been shown that dietary calcium and phosphorus have significant effects on calbindin-D28k expression in the duodenum of broiler chickens [7]. These findings suggest that NaP-IIb, calbindin-D28k and vitamin D receptor expression could be involved in the regulation of calcium and phosphorus absorption.

The objective of the present study was to investigate the laying performance and correlation of intestinal sodium phosphorus co-transporter, calbindin and vitamin D receptor mRNA expression in laying hens with dietary nonphytate phosphorus allowance, and estimate the phosphorus requirement for Dwarf pink-shell laying hens during peak egg production.

## Materials and methods

### Experimental design and diets

The study protocol was approved and conducted in accordance with the Animal Ethics Committee guidelines of China Agricultural University.

A total of 405 Nongda-3 Dwarf pink-shell laying hens with the dwarf gene aged 28 wk old were selected based upon similar body weight of 1.52 ± 0.04 kg/bird and randomly assigned to five dietary treatments. Each treatment consisted of nine replicate pens, nine hens per replicate. All hens were raised in battery cages equipped with an individual feeder and water supply in an environmentally controlled room. Birds were allowed to adapt to experimental diets in the layer house for 2 wk; the entire experiment lasted for 12 wk. According to the assigned treatment group, layers were fed with corn-soybean meals containing either 0.20, 0.25, 0.30, 0.35, or 0.40% nonphytate phosphorus, and had free access to mash feed and water. Diets were formulated on a nonphytate phosphorus basis instead of a total phosphorus basis, because laying hens are unable to absorb phytate phosphorus present in corn and soybean meal. Nutrients (except phosphorus) meet the requirements recommended by the Chinese Chicken Feeding Standard Requirements (NY/T 33–2004). Phosphorus in the feed was analyzed using a vanadate-molybdate reagent [8]. Ingredients and nutrient composition of the experimental diets are shown in Table 1. Performance of each laying hen was monitored and eggs were collected on a daily base. Egg production and feed consumption of each replicate were recorded weekly. Egg production rate, average egg weight, average daily egg production, feed intake and feed/egg ratio were calculated every 4 wk.

**Table 1:** Composition of laying hens diets

**Items**	**Dietary nonphytate phosphorus density, %**				
	**0.20**	**0.25**	**0.30**	**0.35**	**0.40**
Ingredient, %					
Corn	60.49	60.31	60.12	59.95	59.75
Soybean meal	27.14	27.17	27.21	27.24	27.27
Soybean oil	0.97	1.03	1.09	1.15	1.21
Limestone	10.12	9.91	9.71	9.49	9.31
Dicalcium phosphate	0.47	0.77	1.06	1.36	1.65
Salt	0.3	0.3	0.3	0.3	0.3
Trace mineral premix^1^	0.2	0.2	0.2	0.2	0.2
Vitamin premix^2^	0.02	0.02	0.02	0.02	0.02
DL-methionine	0.17	0.17	0.17	0.17	0.17
Choline chloride	0.12	0.12	0.12	0.12	0.12
Total	100	100	100	100	100
Nutrient composition, %					
ME, Mcal/kg	2.7	2.7	2.7	2.7	2.7
CP	17	17	17	17	17
Calcium	3.75	3.75	3.75	3.75	3.75
Nonphytate phosphorus^3^	0.2	0.25	0.3	0.35	0.4
Lysine	0.86	0.86	0.86	0.86	0.86
Methionine + Cystine	0.65	0.65	0.65	0.65	0.65
Tryptophan	0.21	0.21	0.21	0.21	0.21
Threonine	0.66	0.66	0.66	0.66	0.66

^1^Mineral premix provided per kg of diet: Mn, 100 mg; Fe, 80 mg; Zn, 75 mg; Cu, 8 mg; I, 0.35 mg; Se, 0.15 mg.

^2^Vitamin premix provided per kg of diet: vitamin A, 12,500 IU; vitamin D_3_, 2,500 IU; vitamin E, 30 IU; vitamin K_3_, 2.65 mg; vitamin B_1_, 2 mg; vitamin B_2_, 6 mg; vitamin B_12_, 0.025 mg; biotin, 0.0325 mg; folic acid, 1.25 mg; pantothenic acid, 12 mg; niacin, 50 mg.

^3^Analyzed levels of nonphytate phosphorus were 0.19, 0.24, 0.31, 0.35, and 0.42 in each nonphytate phosphorus density diet.

### Tissue sampling and preparation

At the end of the trial, hens were killed by cervical dislocation and duodenum segments (from the gizzard to the bile duct) were removed. Duodenal mucosa was scraped off at the center of individual duodenum segments with a glass microscope slide on ice and immediately frozen in liquid nitrogen for later determination of protein and mRNA expression.

### Test of egg quality

At 41 wk of age, egg quality parameters were checked for a total of 27 eggs from each treatment. Egg shape index was calculated by diameter/height × 100. After breakout, albumen and yolk were separated and weighed. Relative weights of albumen and yolk were calculated against egg weight. The albumen height was measured using a digital micrometer head IP54 (Swiss Precision Instruments, Inc., Garden Grove, California, USA). Yolk color was assessed using the Roche yolk color fan. Haugh units were calculated as described by Haugh [9]. Eggshell breaking strength was determined based upon the vertical axis measured by an Instron 3360 apparatus (Instron, Canton, MA, USA). Eggshells were weighed. Shell thickness was determined at the sharp and blunt ends and equator after removing the shell membranes using a micrometer.

### Total RNA extraction, reverse transcription, and real-time quantitative PCR

Total RNA was extracted from the duodenal mucosa using SV Total RNA Isolation System instructions (Z3100, Promega, Madison, WI, USA). Concentrations of RNA were measured by absorbance at 260 nm, and the integrity of RNA was checked by agarose gel electrophoresis.

Reverse transcription of total RNA to cDNA was conducted with an Avian Myeloblastosis Virus Reverse Transcriptase kit (Promega) in the presence of recombinant RNase in Ribonuclease Inhibitor (A3500, Promega). In total, 1 μg RNA was used and Oligo (dT) 15 was used as the primer.

Gene expression of intestinal type IIb sodium phosphorus co-transporter, calbindin, and vitamin D receptor was determined by real-time quantitative PCR with *β-actin* as the internal standard. The primers used and information of PCR products are listed in Table 2. Real-time PCR was conducted using an ABI 7500 Fluorescent Quantitative PCR System (Applied Biosystems, Bedford, MA, USA) and RealSuper Mixture (with Rox) Kits (CW0767; CWbio, Beijing, China). PCR cycling was as follows: 95°C for 4 min; 40 cycles of 95°C for 15 s, 60°C for 60 s; and 60–95°C for melting curve analysis. Each sample was amplified in triplicate and amplification efficiency was determined by standard curves to ensure equal efficiency between target genes and the internal control standard. The 2–ΔΔCt method was used to calculate the expression of the target gene, as previously described [10].

**Table 2:** Oligonucleotide PCR primers used for the determination of vitamin D receptor protein and sodium phosphate type-IIb mRNA expression

**Gene**	**GenBank accession**	**Orientation**	**Primer sequence (5′ → 3′)**	**Predicted size, bp**
Sodium Phosphate type-IIb	NM_204474.1	Forward	CTTTTACTTGGCTGGCTGGAT	148
		Reverse	AGGGTGAGGGGATAAGAACG	
Calbindin	NM_205513.1	Forward	AATCTGCGTTGCTTCCATACA	218
		Reverse	CATTTAGTTGCCTGAGTTCACCT	
Vitamin D receptor	NM_205098.1	Forward	GGCTCAGGTTTTGCAGATTTG	100
		Reverse	CAGCATCGCCTTTCCCATT	
*β-actin*	NM_205518.1	Forward	AACACCCACACCCCTGTGAT	100
		Reverse	TGAGTCAAGCGCCAAAAGAA	

### Quantification of intestinal calbindin and vitamin D receptor proteins

Duodenum mucosa was homogenized by sonication using an Ultrasonic Cell Disruption System (JY99-IIIB; Ningbo Scientz Biotechnology Co. Ltd, Ningbo, China). Homogenates were centrifuged at 10,000 × *g* for 5 min at 4°C and supernatants were collected and stored at −80°C. The total protein content in each supernatant was determined by using the Bradford method [11]. Western blot analysis was performed as described by Li *et al*. [12]. Band densities were analyzed using AlphaEase Stand Alone Software (Alpha Innotech, Santa Clara, CA, USA), as described by Guo *et al*. [13].

### Tibia strength, tibia ash, calcium and phosphorus concentration

Tibias were directly de-fleshed and patellas were removed. Tibia breaking strengths of fresh bone were measured using a WDS-1 electric universal testing machine by three-point bending test of metaphyseal tibia with 30-mm supporting distance and 10-mm/min test speed. Tibias were air dried for 24 h at room temperature. They were defatted, dried at 105°C for 24 h and placed in a desiccator. Bone weight was recorded. Tibias were ashed at 550°C for 16 h to determine the percentages of ash, phosphorus and calcium. Phosphorus and calcium content were determined by ammonium metavanadate colorimetric and EDTA titration methods, respectively [14].

### Statistical analysis

For all statistical analyses, each replicate served as the experimental unit. All data were analyzed using the GLM procedure in SPSS 16.0 to estimate the main effects. A *P* < 0.05 was considered statistically significant.

## Results

### Performance of laying hens

The results of the performance of the laying hens are provided in Table 3. The feed intake for treatment with dietary nonphytate phosphorus at the level of 0.2% was significantly lower than that for the other treatments (*P* < 0.01). The hen-day egg production of laying hens fed with 0.3% nonphytate phosphorus was significantly higher than laying hens fed with 0.2% and 0.4% nonphytate phosphorus (*P* < 0.05). No difference was observed in egg production and feed conversion ratio (feed/egg) of laying hens. Average egg weight, hen-day egg production and feed intake, however, were significantly influenced by dietary nonphytate phosphorus (*P* < 0.05). The highest average egg weight, hen-day egg production and feed intake were predicted to be obtained at the nonphytate phosphorus level of 0.3% based on quadratic relationships with dietary nonphytate phosphorus content (*P* < 0.05).

**Table 3:** **Effect of nonphytate phosphorus levels on productivity of laying hens from 30 to 41 wk of age**^**1**^

**Nonphytate phosphorus, %**	**Egg production, %**	**Egg weight, g**	**Feed/egg ratio**	**Feed intake, g**	**Hen-day egg production, g**	**Broken egg, %**	**Mortality, %**
0.20	87.25	51.33^b^	2.10	91.45^b^	44.81^b^	0.26	0.012
0.25	90.95	51.72^ab^	2.10	96.37^a^	46.74^ab^	0.16	0.049
0.30	90.44	52.67^a^	2.11	97.06^a^	47.63^a^	0.18	0.049
0.35	88.03	52.67^a^	2.14	95.63^a^	46.40^ab^	0.16	0.012
0.40	88.77	51.62^ab^	2.13	94.5^a^	45.29^b^	0.18	0.037
SEM	0.49	0.18	0.01	0.53	0.34	0.03	0.010
*P*-value	0.075	0.037	0.887	0.002	0.035	0.823	0.608
Linear	0.893	0.210	0.335	0.092	0.773	0.892	0.446
Quadratic	0.045	0.010	0.975	0.000	0.002	0.987	0.364

^a, b^Within comparisons, means in a column with no common superscripts differ significantly (*P* < 0.05).

^1^Nine replications per treatment, three cages per replication, three hens per cage.

### Egg quality

Egg quality did not differ among all treatment groups (Table 4 and 5). Increased dietary nonphytate phosphorus tended to increase the percentage of eggshell (*P* = 0.051) and eggshell strength (*P* = 0.059). The colors of the egg yolk linearly increased as the dietary levels of nonphytate phosphorus increased (*P* < 0.05). The relative percentage of albumen tended to correlate with dietary nonphytate phosphorus quadratically (*P* < 0.05). The highest percentage of albumen was observed with nonphytate phosphorus at 0.25%.

**Table 4:** Effects of nonphytate phosphorus levels on egg quality of laying hens fed at 41 wk of age

**Nonphytate phosphorus, %**	**Percentage of yolk, %**	**Percentage of albumen, %**	**Percentage of eggshell, %**	**Eggshell strength**, **kg/cm**^**2**^	**Eggshell thickness, mm**
0.20	28.88	57.28	13.84	3.34	0.334
0.25	27.78	58.69	13.53	3.05	0.330
0.30	28.04	58.32	13.61	3.42	0.336
0.35	28.03	57.49	14.48	3.38	0.338
0.40	28.29	57.46	14.24	3.75	0.342
SEM	0.14	0.20	0.12	0.07	0.002
*P*-value	0.137	0.068	0.066	0.095	0.476
Linear	0.339	0.537	0.051	0.059	0.115
Quadratic	0.044	0.037	0.361	0.116	0.501

**Table 5:** Effects of nonphytate phosphorus levels on egg quality of laying hens fed at 41 wk of age

**Nonphytate phosphorus, % **	**Albumen height, mm**	**Egg shape index**	**Yolk color**	**Shell color**	**Haugh units**
0.20	6.57	1.337	7.92	7.91	81.67
0.25	6.59	1.337	8.24	8.24	80.73
0.30	6.93	1.341	8.31	8.31	86.04
0.35	6.82	1.345	8.58	8.58	84.24
0.40	6.99	1.344	8.68	8.68	85.28
SEM	0.11	0.003	0.06	0.06	0.59
*P*-value	0.659	0.703	0.058	0.352	0.759
Linear	0.180	0.807	0.008	0.074	0.246
Quadratic	0.816	0.615	0.291	0.922	0.962

### Gene expression of NaP-IIb, calbindin and vitamin D receptor

Data of gene expression of NaP-IIb, calbindin, and vitamin D receptor are shown in Table 6. Dietary nonphytate phosphorus significantly affected the expression of NaP-IIb, vitamin D receptor and calbindin mRNA in the duodenum mucosa of laying hens after 12 wk (*P* < 0.01). At 41 wk of age, the expression of NaP-IIb in the duodenum decreased as dietary phosphorus increased from 0.2 to 0.4%, while duodenal expression of calbindin and vitamin D receptor quadratically correlated with the level of phosphorus (*P* < 0.01). When the dietary nonphytate phosphorus level ranged from 0.20 to 0.40%, the highest NaP-IIb mRNA expression was observed in laying hens fed dietary nonphytate phosphorus at 0.20%, and the lowest was in laying hens when fed dietary nonphytate phosphorus at 0.40%. Vitamin D receptor mRNA expression in the 0.25% group was higher (*P* < 0.01) than the other groups. The highest abundance of vitamin D receptor and calbindin was observed in laying hens fed dietary nonphytate phosphorus at 0.25%.

**Table 6:** Effects of different dietary phosphorus levels on gene expression of duodenal NaP-IIb, calbindin, vitamin D receptor, and protein abundance of calbindin and vitamin D receptor of laying hens at 41 wk of age

**Nonphytate phosphorus, %**	**Duodenal NaP-IIb mRNA**^**1**^	**Duodenal calbindin mRNA**^**1**^	**Duodenal vitamin D receptor mRNA**^**1**^	**Duodenal calbindin protein**^**2**^	**Duodenal vitamin D receptor protein**^**2**^
0.20	1.16^a^	0.96^c^	1.37^b^	0.21^b^	0.17^d^
0.25	0.76^b^	26.23^a^	3.17^a^	0.29^b^	0.27^c^
0.30	0.64^b^	16.30^a^	1.43^b^	0.72^a^	0.62^a^
0.35	0.65^b^	17.40^a^	0.94^b^	0.49^a^	0.49^b^
0.40	0.26^c^	2.49^b^	1.27^b^	0.21^b^	0.19^d^
SEM	0.054	2.29	0.160	0.05	0.04
*P*-value	0.000	0.000	0.000	0.000	0.000
Linear	0.000	0.684	0.000	0.036	0.002
Quadratic	0.678	0.000	0.000	0.000	0.000

^a–d^Within comparisons, means in a column with no common superscript differ significantly (*P* < 0.05) (n = 5).

^1^mRNA relative expression is normalized to *β-actin*, results are expressed as 2-△△Ct.

^2^Relative optical densityof protein to *β-actin*.

### Protein abundance of duodenal calbindin and vitamin D receptor

In the duodenum, the abundance of calbindin and vitamin D receptor in the mucosa was influenced remarkably by dietary nonphytate phosphorus (*P* < 0.01) (Table 6). Both duodenal calbindin and vitamin D receptor displayed significant quadratic relationships with dietary nonphytate phosphorus content after 12 wk of feeding. The highest abundance of both proteins’ expression occurred when dietary nonphytate phosphorus was at 0.3%.

### Tibia mineral composition and breaking strength

Mineral composition and breaking strength of tibia in broiler chickens fed different dietary nonphytate phosphorus levels are shown in Table 7. Increased dietary nonphytate phosphorus tended to quadratically increase tibia ash content (*P* = 0.070) and tibia phosphorus content (*P* = 0.073), with the highest ash and phosphorus contents at the level of 0.3% dietary nonphytate phosphorus. There was no correlation between nonphytate phosphorus in the diet and tibia breaking strength and tibia calcium content.

**Table 7:** Mineral composition and breaking strength of hentibias influenced by different dietary phosphorus levels

**Nonphytate phosphorus, %**	**Tibia ash**^**1**^**, %**	Tibia phosphorus*, %	Tibia calcium*, %	**Tibia breaking strength, N**
0.20	47.85	16.05	36.12	182.58
0.25	50.67	16.29	36.21	180.66
0.30	51.21	16.75	36.98	231.18
0.35	50.54	16.44	36.17	213.08
0.40	49.79	16.06	35.36	196.88
SEM	0.53	0.13	0.36	7.40
*P*-value	0.295	0.411	0.786	0.176
Linear	0.330	0.857	0.577	0.229
Quadratic	0.070	0.073	0.303	0.114

^1^Results are expressed on a dry, defatted weight basis of tibia.

*Results are expressed on an ash weight basis of tibia.

## Discussion

Dietary nonphytate phosphorus significantly affected laying performance and feed intake in laying hens during the 12-week experimental period. The expression of NaP-IIb linearly decreased when phosphorus increased in diets. These data suggest a negative feedback effect of dietary phosphorus on its transporters. Quadratic relationships were found between dietary nonphytate phosphorus and average egg weight, egg production, feed intake, and gene and protein expression of vitamin D receptor and calbindin. The highest value was found in laying hens fed a diet with nonphytate phosphorus at the levels of 0.25 to 0.3%.

The estimated nonphytate phosphorus requirements for layers by National Research Council [15] were lowered from 350 mg/hen/d in 1984 to 250 mg/hen/d. Boling *et al*. [16] and Keshavarz [17] reported that no influence on egg production was observed in breeder or laying hens when fed low levels of nonphytate phosphorus. Summers [18] showed that layers fed a maize-soybean meal diet containing 0.2% nonphytate phosphorus performed similarly to laying hens fed a diet containing 0.4% nonphytate phosphorus up to 32 wk; however, egg production was significantly reduced by the lower dietary phosphorus afterwards. Nys *et al*. [19] reported that nonphytate phosphorus at 0.3% maintained normal performance and bone integrity of hens. Ahmadi and Rodehutscord conducted a meta-analysis to conclude the dietary nonphytate phosphorus requirements in laying hens based upon 12 experiments between 1999 and 2011 [20]. Using egg production, egg mass and feed conversion ratio as models, 0.22% of nonphytate phosphorus without phytase supplementation resulted in the highest production performance in laying hens [20]. In contrast, according to the present work, Dwarf pink-shell laying hens could have optimal laying performance when dietary nonphytate phosphorus is set at 0.3%. No differences in shell quality were observed either in the present study or in other studies [16,17,21]. The data suggest that dietary phosphorus at 0.2 to 0.4% may not affect eggshell quality. The difference in dietary nonphytate phosphorus requirements between Dwarf pink-shell laying hens and western commercial layers could come from native characteristics of low feed intake but high egg production.

To estimate the dietary nonphytate phosphorus requirement, egg production, egg mass and feed conversion ratio as well as bone quality are commonly used in model analyses. The NaP-IIb co-transporter is the only known protein that mediates phosphorus transport through the apical membrane of intestinal epithelial cells. Dietary nonphytate phosphorus levels are one of the main factors affecting the function of NaP-IIb in pigs [22]. Low phosphorus intake can stimulate the uptake of phosphorus. Intestinal transport of phosphorus was dependent on NaP-IIb protein and NaP-IIb mRNA expression [23]. In the present study, laying hens fed with dietary nonphytate phosphorus at 0.2% had significantly higher NaP-IIb mRNA expression than laying hens fed with nonphytate phosphorus at 0.4%. This could be a molecular mechanism of laying hens to keep homeostasis of phosphorus by reducing the uptake of phosphorus in the gut when dietary nonphytate phosphorus exceeds requirements.

1,25-dihydroxyvitamin D3 is a critical factor for intestinal phosphate absorption [24]. Tibia ash, tibia strength and intestinal NaP-IIb mRNA expression could be increased by dietary 1α-hydroxycholecalciferol [10]. NaP-IIb mRNA was transcriptionally regulated by vitamin D in the gut [4]. Vitamin D_,_ its metabolites, and also their receptors, could be involved in the regulation of phosphorus absorption in the gut by affecting NaP-IIb mRNA expression. In the present study, the expression of vitamin D receptor mRNA and protein displayed a quadratic correlation with dietary nonphytate phosphorus. Calbindin acts as a dynamic Ca^2+^ buffer, in Ca^2+^ transporting epithelia, and displays an important role in Ca^2+^ induced signal transmission [6]. Calbindin mediates the impact of dietary calcium on phosphorus absorption. In broiler chickens, calbindin mRNA was affected by both calcium and dietary nonphytate phosphorus [12]. In the present study, all diets of the different treatments had the same level of calcium. However, the expression of calbindin at the transcriptional and protein levels changed when dietary nonphytate phosphorus was altered. These data suggest that calbindin might regulate phosphorus absorption independent of dietary calcium.

The level of dietary phosphorus was related to the incidence of tibial dyschondroplasia [25,26]. Tibial dyschondroplasia is the most common skeletal anomaly associated with fast growth in numerous bird species, the result of which is the occurrence of bone deformation and lameness [27]. Tibia with the highest ash and phosphorus content was at the level of 0.3% dietary nonphytate phosphorus. Previous studies with broiler breeds showed that tibia parameters were affected by nonphytate phosphorus levels in the diet [28].

In conclusion, the optimal dietary nonphytate phosphorus allowance for Dwarf pink-shell laying hens is suggested to be 0.3% without phytase supplementation, which is comparable to National Research Council standards [15], but higher than the 0.25% nonphytate phosphorus level suggested by recent publications. High dietary nonphytate phosphorus can decrease intestinal gene expression of sodium phosphorus co-transporters to prevent excess phosphorus absorption in layer hens. With 0.3% nonphytate phosphorus supplementation, calbindin and vitamin D receptor reached their highest expression.

## Competing interests

The authors declare that they have no competing interests.

## Authors’ contributions

WN carried out the experiment, participated in data analysis, and drafted the manuscript. YY, JY and ZW participated in the design of the experiments and conducted statistical analysis. YG conceived the experiment and finished the manuscript. All authors approved the final version of the manuscript for publication.
